# Supplementary material for: Impact of sleep duration on executive function and brain structure
Source: Commun Biol. 2022 Mar 3;5:201. doi: 10.1038/s42003-022-03123-3 (PMC8894343; doi:10.1038/s42003-022-03123-3)
Supplement: Supplementary file 2 — Supplementary Information [file 42003_2022_3123_MOESM2_ESM.pdf]

Supplementary material

Supplementary Table 1| Population demographics

Supplementary Table 2| Biobank showcase variables used in all analyses

Supplementary Figure 1 | Relationship between sleep duration and individual cognitive tasks

Supplementary Table 3 | Association between summary volume measure of sleep-related brain regions with sleep duration, sleep characteristics, obstructive sleep apnoea traits and baseline characteristics of vascular status, genetic risk and socioeconomic status.

Supplementary Figure 2 | Differences in brain volume across 139 cortical and subcortical brain regions in participants who slept between six-to-eight hours and other sleep durations.

Supplementary Figure 3 | Differences in brain volume across 139 cortical and subcortical brain regions in participants who slept between six-to-eight hours and more than eight hours duration.

Supplementary Figure 4 | Differences in brain volume across 139 cortical and subcortical brain regions in participants who slept between six-to-eight hours and less than eight hours duration.

Supplementary Figure 5 | Regional brain volumes which are significantly associated with a higher executive function score and sleep duration six-to-eight hours.

Supplementary Figure 6 | Boxplots representation of data from main paper figures.

Supplementary Table 1 | Population demographics

**Total N**	479 420
**Sex**	
**Number Female (%)**	262 777 (54.8)
**Number Male (%)**	216 643 (45.2)
**Mean age at baseline assessment (SD)**	57.4 (8.0)
**Mean age at Imaging assessment (SD)**	65.1 (7.7)
**Median Townsend Index (IQR)**	-2.2 (3.1)
**Median sleep duration in hours (range)**	7 (1-22)
<u>Medication at baseline assessment:</u>	
**Blood pressure medication**	
**ON (%)**	28 894 (13.4)
**Cholesterol lowering medication**	
**ON (%)**	38 069 (17.7)
**Diabetes medication**	
**ON (%)**	1659 (1.0)
**Smoking status**	
**Number never smoked (%)**	369 906 (77.2)
**Number 1-20 pack years (%)**	63 461 (13.2)
**Number >20 pack years (%)**	46 054 (9.6)
***APOE* ε Status**	
**Number of ε3/3 (%)**	230 829 (69.4)
**Number of ε3/4 (%)**	92 716 (27.9)
**Number of ε4/4 (%)**	9314 (1.9)
**Mean Body mass Index (SD)**	27.4 (4.8)
**Male**	27.8 (4.2)
**Female**	27.1 (5.2)

SD: standard deviation; IQR: interquartile range; APOE: apolipoprotein E.

Supplementary Table 2. Biobank showcase for information for variables

Variable	Field ID	Biobank showcase link	Instance
**Year of birth**	34	https://biobank.ndph.ox.ac.uk/showcase/field.cgi?id=34	0
**Date of attending assessment centre**	53	https://biobank.ndph.ox.ac.uk/showcase/field.cgi?id=53	0,2
**Sleep duration**	1160	https://biobank.ndph.ox.ac.uk/showcase/field.cgi?id=1160	0, 2
**Sleep chronotype**	1180	https://biobank.ndph.ox.ac.uk/showcase/field.cgi?id=1180	0,2
**Report of sleeplessness/ insomnia**	1200	https://biobank.ndph.ox.ac.uk/showcase/field.cgi?id=1200	0,2
**Report of snoring**	1210	https://biobank.ndph.ox.ac.uk/showcase/field.cgi?id=1210	0,2
**Report of daytime sleepiness**	1220	https://biobank.ndph.ox.ac.uk/showcase/field.cgi?id=1220	0,2
**Reaction time**	20023	http://biobank.ndph.ox.ac.uk/showcase/field.cgi?id=20023	0
**Pairs matching accuracy**	399	http://biobank.ndph.ox.ac.uk/showcase/field.cgi?id=399	0
**Trail making numeric path duration**	6348	https://biobank.ndph.ox.ac.uk/showcase/field.cgi?id=6348	2
**Trail making alphanumeric path duration**	6350	https://biobank.ndph.ox.ac.uk/showcase/field.cgi?id=6350	2
**Tower Rearranging number of puzzles correct**	21004	https://biobank.ndph.ox.ac.uk/showcase/field.cgi?id=21004	2
**Tower Rearranging number of puzzles attempted**	6383	https://biobank.ndph.ox.ac.uk/showcase/field.cgi?id=6383	2
**Symbol-digit substitution number correct**	23323	https://biobank.ndph.ox.ac.uk/showcase/field.cgi?id=23323	2
**Symbol-digit substitution number attempted**	23324	https://biobank.ndph.ox.ac.uk/showcase/field.cgi?id=23324	2
**Townsend deprivation index (at recruitment)**	189	https://biobank.ndph.ox.ac.uk/showcase/field.cgi?id=189	0
**Cholesterol lowering medication**	6177	http://biobank.ctsu.ox.ac.uk/showcase/field.cgi?id=6177	0
**Blood pressure medication**	6177	http://biobank.ctsu.ox.ac.uk/showcase/field.cgi?id=6177	0
**Diabetes medication**	6177	http://biobank.ctsu.ox.ac.uk/showcase/field.cgi?id=6177	0

**Body Mass Index**	23104	https://biobank.ndph.ox.ac.uk/showcase/field.cgi?id=23104	0,2
**Number of cigarettes smoked daily (current smokers)**	2887	http://biobank.ctsu.ox.ac.uk/crystal/field.cgi?id=2887	0
**Number of cigarettes smoked daily (previous smokers)**	6183	http://biobank.ctsu.ox.ac.uk/crystal/field.cgi?id=6183	0
**Age started smoking (current smokers)**		http://biobank.ctsu.ox.ac.uk/crystal/field.cgi?id=3436	0
	3436		
**Age started smoking (former smokers)**	2867	http://biobank.ctsu.ox.ac.uk/crystal/field.cgi?id=2867	0
**Ever tried to stop smoking**	3486	http://biobank.ctsu.ox.ac.uk/crystal/field.cgi?id=3486	1
**Stopped smoking (>6 months)**	2907	http://biobank.ctsu.ox.ac.uk/crystal/field.cgi?id=2907	1
**Age stopped smoking**	2897	http://biobank.ctsu.ox.ac.uk/crystal/field.cgi?id=2897	1
**Regional grey matter volumes (FAST)**	1101	https://biobank.ndph.ox.ac.uk/showcase/label.cgi?id=1101	2
**Subcortical volumes (FIRST)**	1102	https://biobank.ndph.ox.ac.uk/showcase/label.cgi?id=1102	2
**White matter hyperintensity volume**	25781	http://biobank.ndph.ox.ac.uk/showcase/field.cgi?id=25781	2

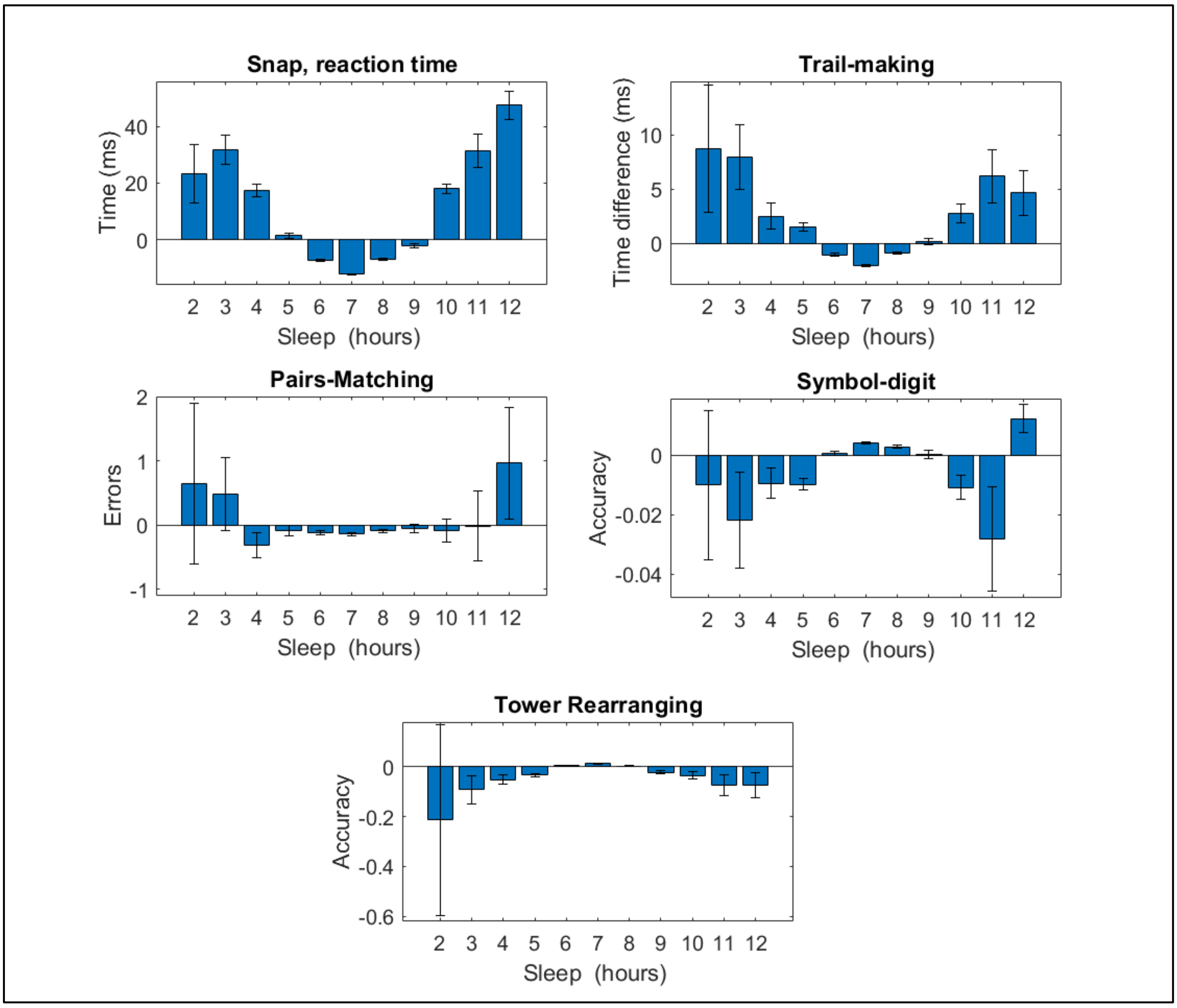

Supplementary Figure 1. Relationship between sleep duration and individual cognitive tasks

The performance on individual cognitive tasks (age-residuals) across different sleep durations showed a quadratic, inverted ‘u’-shape relationship with better performance associated with seven hours of sleep, as seen with the latent variable of executive function, in all cognitive tasks apart from pairs-matching.

Supplementary Table 3. Association between volume of sleep-related brain regions with sleep duration (6-8 hours), sleep characteristics, obstructive sleep apnoea traits and baseline characteristics of vascular status, genetic risk and socioeconomic status.

	Unstandardized beta estimate	Standard Error	t	p
**Sleep band 6-8 hours**	**0.063**	**0.028**	**2.24**	***0.025**
Sleep chronotype				
Intermediate (baseline)	--	--	--	--
Morning	0.021	0.021	1.00	0.316
Evening	-0.028	0.030	-0.94	0.346
Report of insomnia	0.010	0.012	0.84	0.400
Obstructive sleep apnoea traits				
Daytime sleepiness	-0.026	0.017	-1.51	0.130
Report of snoring	-0.049	0.018	-2.66	***0.008**
Smoking pack years	-0.003	0.001	-5.05	***<0.001**
Vascular comorbidity	-0.054	0.013	-4.31	***<0.001**
APOE ε status	-0.001	0.017	-0.05	0.957
Body mass index (BMI)	-0.021	0.003	-7.91	***<0.001**
Socio-economic status	-0.005	0.003	-1.39	0.164

Summary volume measure was created based on 46 brain regions which had a significant quadratic relationship with sleep duration.

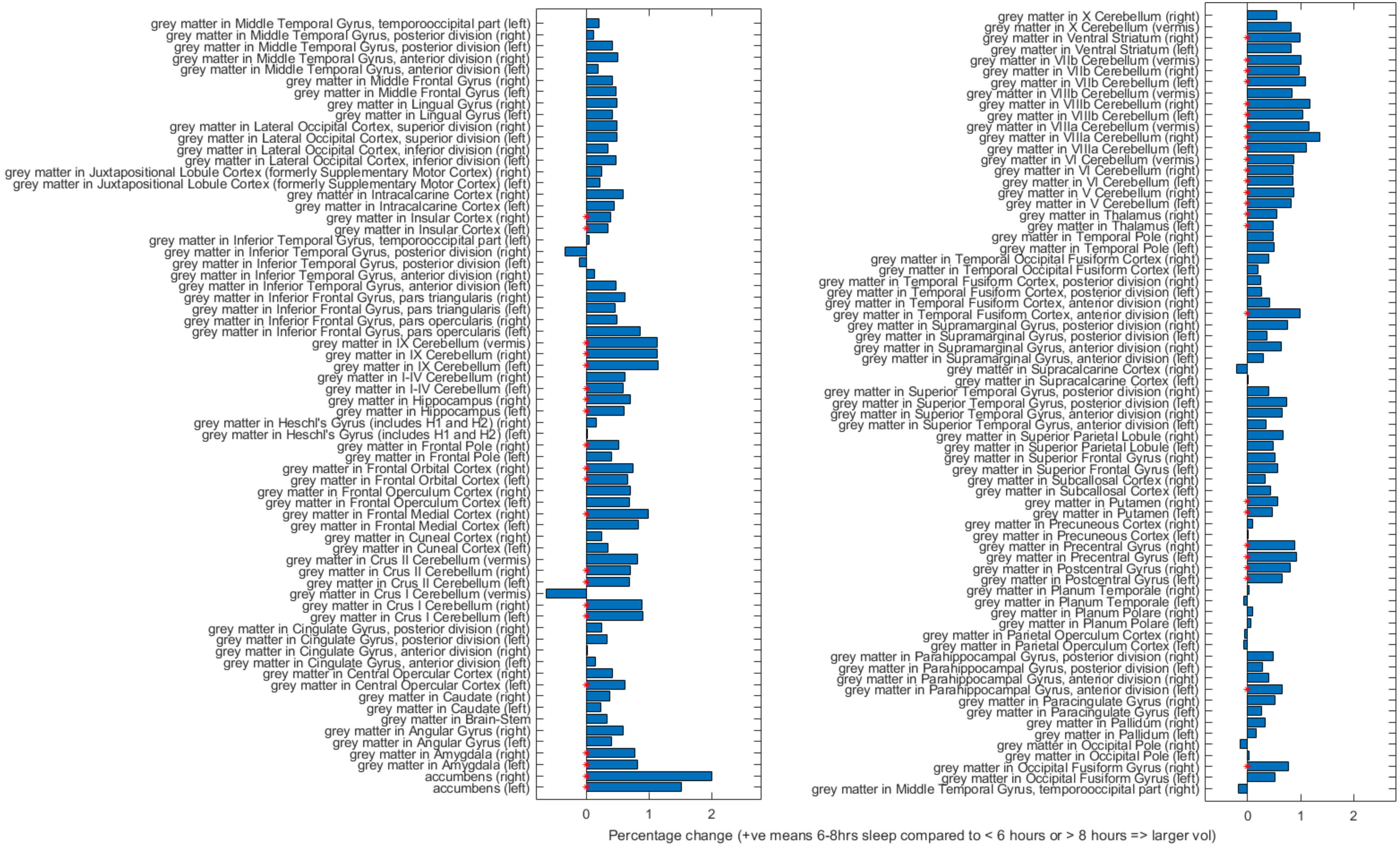

Supplementary Figure 2. Differences in brain volume across 139 cortical and subcortical brain regions in participants who slept between six-to-eight hours and all other sleep duration. Red stars (*) indicates brain volumes with significant difference, following multiple comparison.

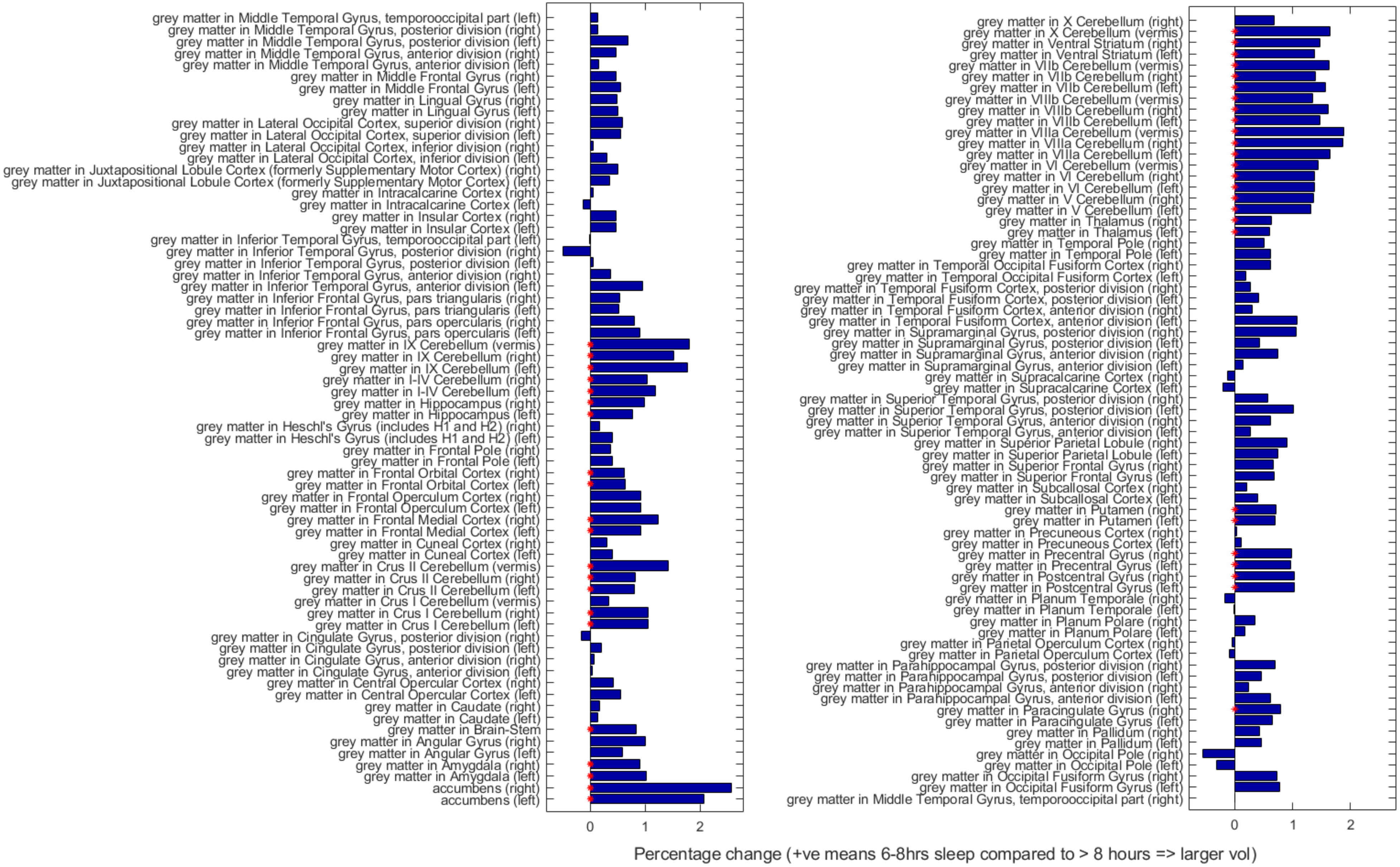

Supplementary Figure 3. Differences in brain volume across 139 cortical and subcortical brain regions in participants who slept between six-to-eight hours and more than eight hours duration. Red stars (*) indicates brain volumes with significant difference, following multiple comparison.

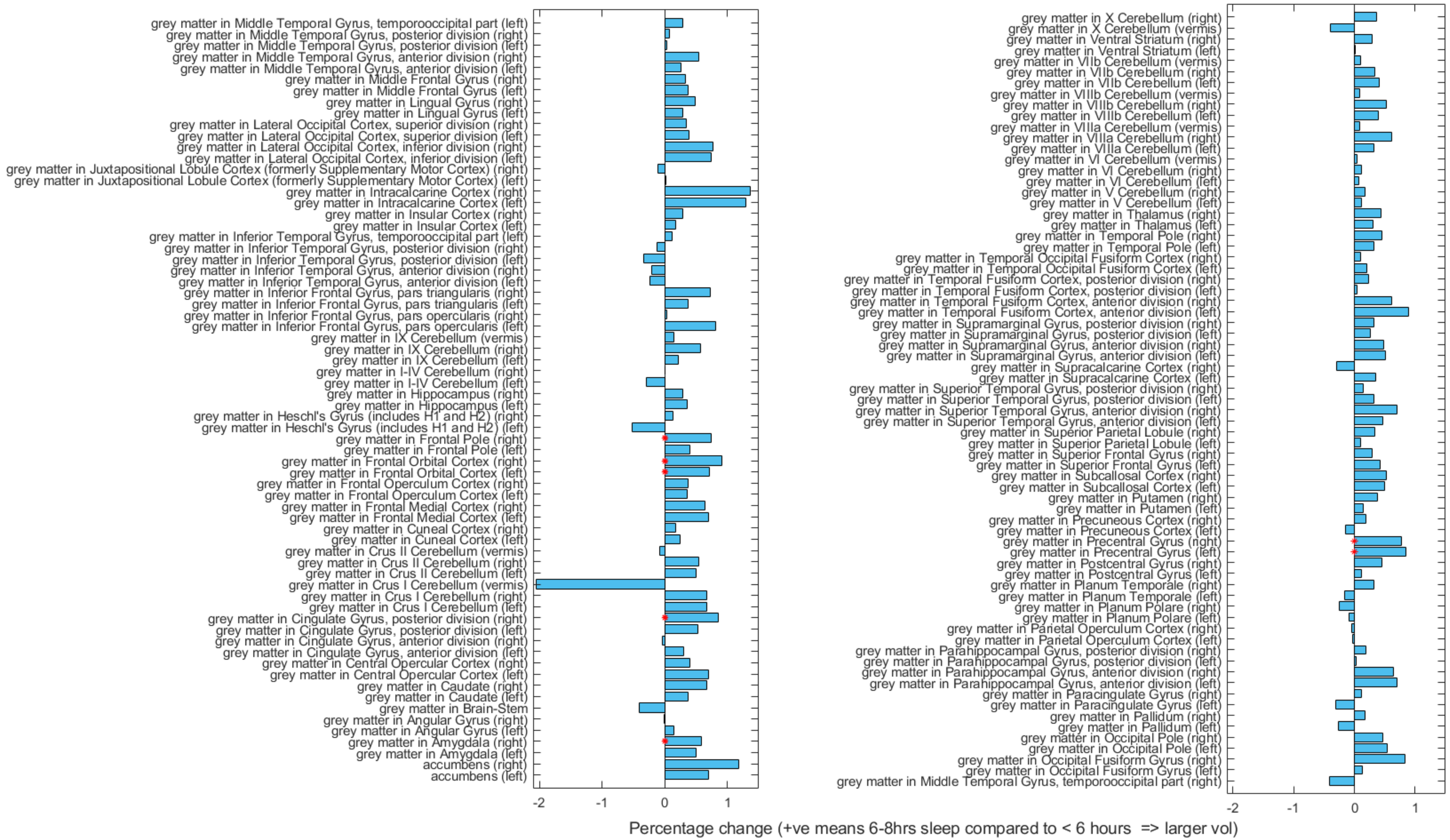

Supplementary Figure 4. Differences in brain volume across 139 cortical and subcortical brain regions in participants who slept between six-to-eight hours and less than eight hours duration. Red stars (*) indicates brain volumes with significant difference, following multiple comparison.

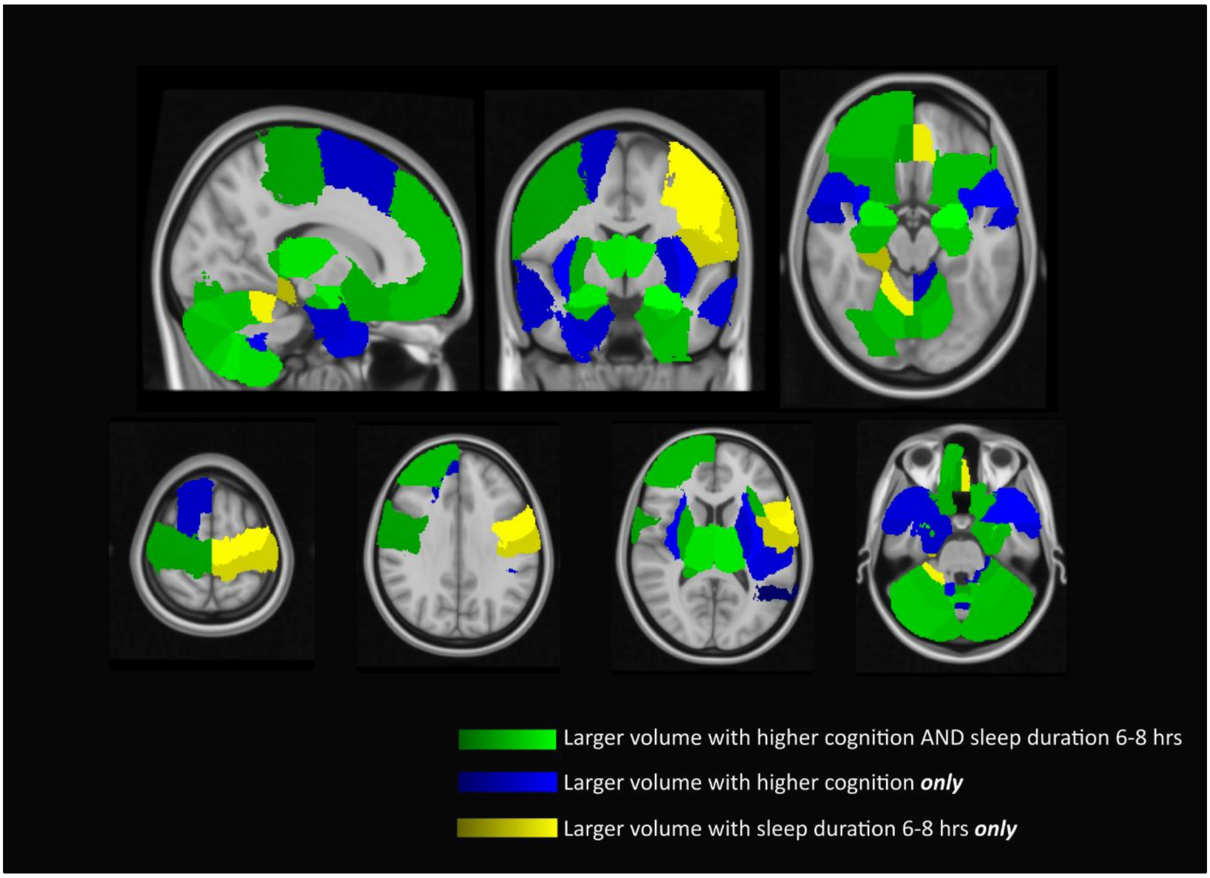

Supplementary Figure 5. Regional brain volumes which are significantly associated with a higher executive function score and sleep duration six-to-eight hours. Volume in 56 brain regions significantly predicted executive function while volume in 46 brain regions was higher with a six-to-eight sleep duration. Forty-one of these brain regions overlapped.

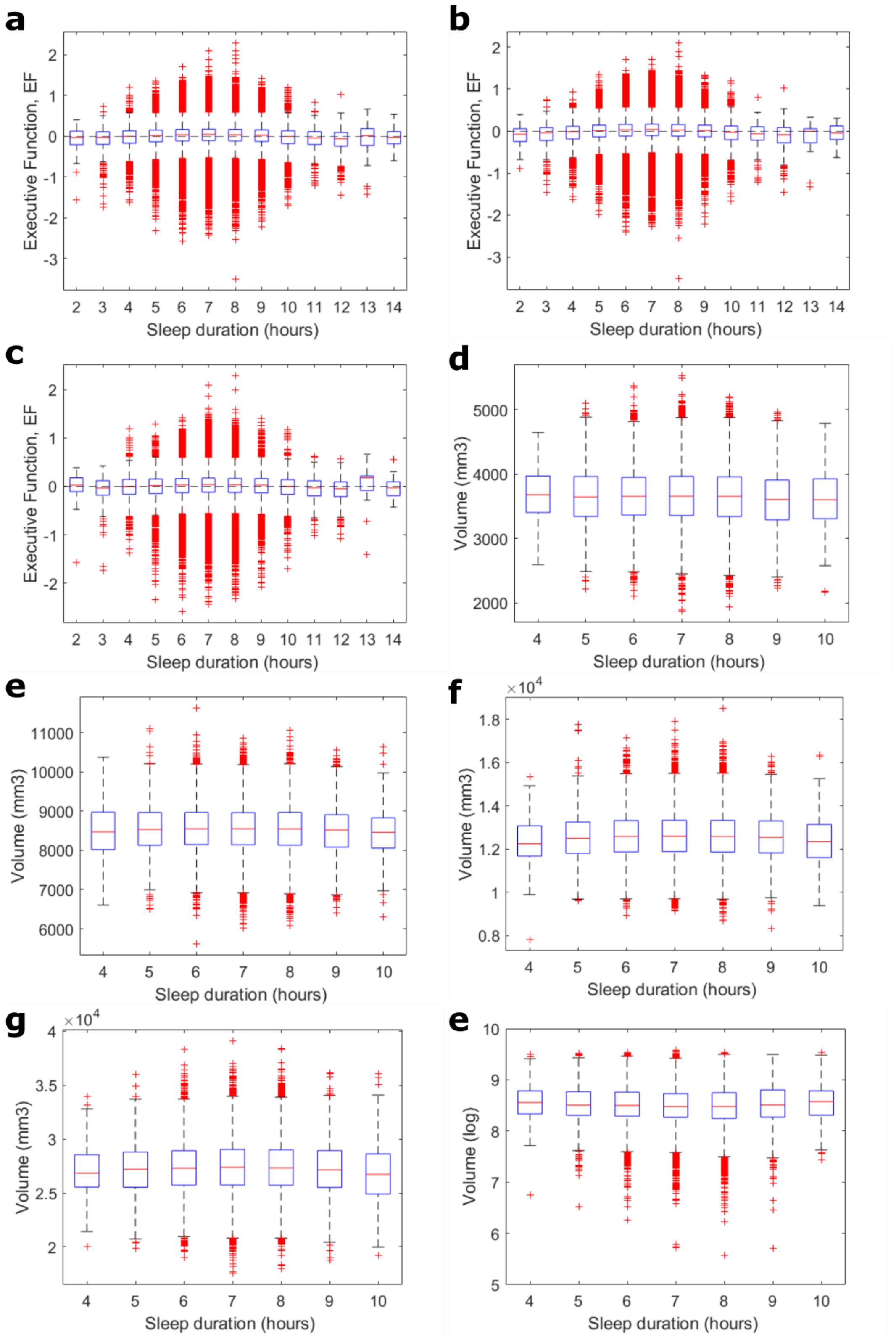

Supplementary Figure 6. Boxplot representations of data from main paper figures. The relationship between sleep duration and age-residual executive function scores in **a,** all participants, **b,** participants younger than 60 years and **c,** participants older than 60 years. Relationship between sleep duration and brain volume in **d,** orbital frontal cortex, **e,** precentral gyrus, **f,** Hippocampi, **g,** Cerebellar vermis regions and **e,** white matter hyperintensities. Centre of each box is the median, bottom and top edges indicate 25th and 75th percentile. Crosses are outliers.
