# Supplementary material for: Impact of sleep duration on executive function and brain structure
Source: Commun Biol. 2022 Mar 3;5:201. doi: 10.1038/s42003-022-03123-3 (PMC8894343; doi:10.1038/s42003-022-03123-3)
Supplement: Supplementary file 3 — Description of Additional Supplementary Files [file 42003_2022_3123_MOESM3_ESM.pdf]

Description of Additional Supplementary Files

File name: Supplementary Data 1
Description: Summary of source data used in article figures.

File name: Supplementary Data 2
Description: Relationship between brain volume in each of the 139 cortical and subcortical brain regions and Executive Function.
